# Evidence-Based Prevention Interventions for People Who Use Illicit Drugs

**DOI:** 10.1155/2013/360957

**Published:** 2013-03-13

**Authors:** Thomas F. Kresina, R. Douglas Bruce, Kevin P. Mulvey

**Affiliations:** ^1^Division of Pharmacologic Therapies, Center for Substance Abuse Treatment, Substance Abuse and Mental Health Services Administration, Rockville, MD 20857, USA; ^2^Yale AIDS Program, Yale University School of Medicine, New Haven, CT 06510, USA; ^3^President's Emergency Plan for AIDS Relief (PEPFAR), U.S. Embassy, 7 Lang Ha Street, Hanoi, Vietnam

The World Drug Report of 2012 [1] reports that, globally, illicit drug use is a common occurrence and is a growing health problem in some regions. Global reporting estimates indicate that approximately 230 million people or 5% of the global adult population have used illicit drugs. While somewhat stable use is ongoing in developed countries, resource-limited settings are seeing the growth and emergency of an illicit drug use as a new and significant national health issue. Heroin, cocaine, and other commonly used illicit drugs are particularly destructive to individual livelihoods, families, and neighborhoods with high social impact and increased morbidity and mortality related to the adverse health consequences of their use [2]. These adverse health consequences include addiction, overdose, violence, mental health disorders, suicide, and infectious complications such as HIV, tuberculosis, and hepatitis C [2–4]. 

To address the adverse health consequences of illicit drug use, the World Health Organization (WHO) and the United Nations Office of Drugs and Crime (UNODC) have developed a comprehensive package of interventions for the prevention and care of HIV among people who inject drugs [5]. There are nine component interventions comprised in this package and they are syringe service programs; medication-assisted treatment and other evidence-based drug dependence treatment; HIV testing and counseling; antiretroviral therapy; prevention and treatment of sexually transmitted infections; condom programs for people who inject drugs and their sexual partners; targeted information, education, and communication for people who inject drugs and their sexual partners; prevention, vaccination, diagnosis, and treatment of viral hepatitis; and prevention, diagnosis, and treatment of tuberculosis. The articles of this special issue address the importance of the prevention of the adverse health consequences of illicit drug use. This issue provides a framework for health policy and model programs across the globe as well as outcomes of the implementation of evidence-based interventions for people who use drugs, particularly in less resource health care systems. 

T. F. Kresina et al. present the importance of a continuum of response as a framework for implementation of evidence-based prevention, care, and treatment interventions as part of a national strategic plan to address HIV/AIDS and the comorbidities associated with injection illicit drug use. This framework is developed by stakeholders who plan, develop, pilot, and provide a full range of services that address the various prevention, care/support, and treatment needs of individuals, families, and communities. 

S. Pinkham et al. provide an overview of the need for multifaceted interventions for women who inject drugs that address relationship dynamics, housing, employment family life, and children's issues and needs as well as improved sexual and reproductive health care for women.

R. Yorick et al. present innovative service models that have been developed and implemented in the Russian Federation and Ukraine that comprise gender-specific approaches to drug rehabilitation, modification of risk behaviors, and psychosocial programs. In an enabling environment, services for women have been delivered and include prenatal care, child-care, women-only programs, mental health services, and workshops on women-focused topics. These service models have specifically reached out to recently released female detainees in the criminal justice system as well as street-involved girls and young women. 

M. V. Volik et al. describe the development of a combination HIV prevention program for people who were opioid dependent that was implemented as a collaboration among the Russian federal government, nongovernment organizations, and civil society. The collaboration resulted in a model of a continuum of care comprised of a recommended package of HIV prevention services for people who inject drugs. The collaborative implementation has resulted in a unique relationship among the government and nongovernment stakeholders as well as program sustainability. 

A. A. Boltaev et al. present an evaluation of the quality and effectiveness of a pilot medication-assisted treatment (MAT) program at three sites in Kazakhstan. The assessment determined the strengths and challenges of the MAT programs as well as the extent to which the MAT programs complied with the minimal recommendations developed by the WHO for psychosocially assisted pharmacologic treatment of opioid dependence. 

E. A. Ratliff et al. introduce the reader to the emerging heroin epidemic in Eastern Africa and how that epidemic contributes to the national HIV/AIDS epidemic. The authors describe how the Tanzania AIDS Prevention Program has collaborated with NGOs and civil society to address heroin abuse as a public health issue with the implementation of MAT programs, syringe service programs, and a recovery program to include sober houses for residential treatment. 

T. T. M. Nguyen et al. take us to Vietnam which is a country that has a high rate of HIV infection among people who inject drugs, as high as 60% in some provinces. Initial efforts to address this public health problem were centered around home and community detoxification programs as well as placement in rehabilitation centers. Recently, MAT programs have been piloted and shown to reduce HIV prevalence among those in treatment while improving quality of life. The authors also present the national scale-up plan for Vietnam. 

L. M. Giang et al. present an integrated approach to preventive health focusing on men who have sex with men (MSM). The approach addresses syndemic conditions including substance use and abuse, mental health, and stigma, all of which play a role in reducing access and participation in prevention health care services. 

B. Myers et al. present an evaluation of an important prevention intervention: screening and brief interventions to address alcohol and other drug use. This intervention was implemented in an emergency room setting by peers. The authors found that approximately 1 in 5 patients utilizing emergency services met the criteria for harmful alcohol or illicit drug use. The authors discuss the barriers to implementation and the importance of addressing substance use disorders in an emergency setting in South Africa. 

E. Buckingham et al. provide a rationale for providing treatment for mental disorders as part of HIV prevention for people who use drugs. The authors discuss the importance of both detection and treatment of mental illness in the context of primary and secondary HIV prevention. The authors found limited models for implementation in low and middle income countries and propose models on integration of services to best address both substance use and cooccurring mental disorders in people who use illicit drugs. 

These papers present a snapshot of the exciting and insightful manner in which prevention interventions are being developed and implemented globally to address the needs of people who use illicit drugs. Barriers to implementation and emerging new models of service delivery are presented in this special issue which is presented with the hope of inspiring further development and scale-up. 

## References

[1] United Nations Office of Drugs and Crime http://www.unodc.org/documents/data-and-analysis/WDR2012/WDR_2012_web_small.pdf.

[2] Degenhardt L, Hall W (2012). Extent of illicit drug use and dependence, and their contribution to the global burden of disease. *The Lancet*.

[3] Nelson P, McLaren J, Degenhardt L (2010). What do we know about the extent of heroin and other opioid use and dependence? results of a global systematic review-NDARC.

[4] World Health Organization (2009). *Global Health Risks: Mortality Ans Burden of Disease Attributable To Selected Major Risks*.

[5] (2012). WHO, UNODC, UNAIDS Technical Guide for countires to set targets for universal access to HIV prevention, treatment and care for injection drug users. 2012 revision.

